# The cytokine tumor necrosis factor-like weak inducer of apoptosis and its receptor fibroblast growth factor-inducible 14 have a neuroprotective effect in the central nervous system

**DOI:** 10.1186/1742-2094-9-45

**Published:** 2012-03-06

**Authors:** Ramiro Echeverry, Fang Wu, Woldeab B Haile, Jialing Wu, Manuel Yepes

**Affiliations:** 1 Department of Neurology and Center for Neurodegenerative Disease, Emory University School of Medicine, Atlanta, GA, USA; 2 Department of Neurology, Tianjin Huanhu Hospital, Tianjin, China; 3 Department of Neurology, Veterans Affairs Medical Center, Atlanta, GA, USA; 4 Department of Neurology and Center for Neurodegenerative Disease, Whitehead Biomedical Research Building, 615 Michael Street, Suite 505J, Atlanta, GA 30322, USA

**Keywords:** TWEAK, Cerebral ischemia, Inflammation, Ischemic tolerance, Preconditioning

## Abstract

**Background:**

Cerebral cortical neurons have a high vulnerability to the harmful effects of hypoxia. However, the brain has the ability to detect and accommodate to hypoxic conditions. This phenomenon, known as preconditioning, is a natural adaptive process highly preserved among species whereby exposure to sub-lethal hypoxia promotes the acquisition of tolerance to a subsequent lethal hypoxic injury. The cytokine tumor necrosis factor-like weak inducer of apoptosis (TWEAK) and its receptor fibroblast growth factor-inducible 14 (Fn14) are found in neurons and their expression is induced by exposure to sub-lethal hypoxia. Accordingly, in this work we tested the hypothesis that the interaction between TWEAK and Fn14 induces tolerance to lethal hypoxic and ischemic conditions.

**Methods:**

Here we used *in vitro *and *in vivo *models of hypoxic and ischemic preconditioning, an animal model of transient middle cerebral artery occlusion and mice and neurons genetically deficient in TWEAK, Fn14, or tumor necrosis factor alpha (TNF-α) to investigate whether treatment with recombinant TWEAK or an increase in the expression of endogenous TWEAK renders neurons tolerant to lethal hypoxia. We used enzyme-linked immunosorbent assay to study the effect of TWEAK on the expression of neuronal TNF-α, Western blot analysis to investigate whether the effect of TWEAK was mediated by activation of mitogen-activated protein kinases and immunohistochemical techniques and quantitative real-time polymerase chain reaction analysis to study the effect of TWEAK on apoptotic cell death.

**Results:**

We found that either treatment with recombinant TWEAK or an increase in the expression of TWEAK and Fn14 induce hypoxic and ischemic tolerance *in vivo *and *in vitro*. This protective effect is mediated by neuronal TNF-α and activation of the extracellular signal-regulated kinases 1 and 2 pathway via phosphorylation and inactivation of the B-cell lymphoma 2-associated death promoter protein.

**Conclusions:**

Our work indicate that the interaction between TWEAK and Fn14 triggers the activation of a cell signaling pathway that results in the induction of tolerance to lethal hypoxia and ischemia. These data indicate that TWEAK may be a potential therapeutic strategy to protect the brain from the devastating effects of an ischemic injury.

## Background

It has been long recognized that cerebral cortical neurons have a high vulnerability to the deleterious effects of hypoxia. However, despite its obvious clinical importance, the development of a successful neuroprotective strategy to protect the brain from the harmful consequences of an ischemic insult has been largely unsuccessful. Preconditioning is a natural adaptive process highly preserved among species whereby a sub-lethal insult (preconditioning event) promotes the acquisition of tolerance to an otherwise lethal environmental change [1]. Accordingly, exposure to a sub-lethal injury, including a short episode of hypoxia and/or ischemia, renders neurons resistant to a subsequent lethal hypoxic or ischemic insult [2]. Because ischemic stroke in the third cause of mortality and a leading cause of disability in the world [3], understanding the mechanisms underlying this phenomenon, known as 'ischemic tolerance', is of the utmost importance for the development of an effective neuroprotective strategy for the treatment of acute ischemic stroke patients.

Tumor necrosis factor-like weak inducer of apoptosis (TWEAK) is a member of the tumor necrosis factor (TNF) superfamily of cytokines [4] that is found in the central nervous system in endothelial cells, perivascular astrocytes, neurons and microglia [5,6]. Fibroblast growth factor-inducible 14 (Fn14) is the receptor for TWEAK [7] and binding of TWEAK to Fn14 has been reported to stimulate cell proliferation [8-10], migration [9-11] and differentiation [12], as well as the expression of pro-inflammatory molecules [4,9,13-17].

Experimental work with animal models of cerebral ischemia [5,18,19] and acute ischemic stroke patients [20] indicates that the onset of the ischemic insult is followed by an increase in the expression of TWEAK and Fn14 in the ischemic tissue and serum, which has been deemed to have a negative impact on the final neurological outcome. Hence, the interaction between TWEAK and Fn14 activates a proinflammatory cell signaling pathway (reviewed in [21]), which has been linked to cell death during cerebral ischemia [22]. Accordingly, a genetic deficiency of TWEAK or Fn14 [23], or treatment with anti-TWEAK neutralizing antibodies [18] or a soluble Fn14-Fc decoy receptor [5] reduces the volume of the ischemic lesion following the induction of experimental ischemic stroke.

It has been reported that TWEAK induces apoptotic cell death in neuronal cultures [18,24]. However, it is known that TWEAK is a poor cytotoxic agent that induces cell death in conjunction with other sensitizers (reviewed in [21]) via multiple mechanisms, including caspase-dependent apoptosis and cathepsin-mediated necrosis [25]. In contrast with these observations, experimental work with glial cell tumors indicate that the interaction between TWEAK and Fn14 has a pro-survival effect mediated by the induction of B-cell lymphoma 2 (Bcl-2) proteins [26].

The cytokine TNF-α is a member of the TNF superfamily of ligands synthesized as a monomeric type-2 transmembrane protein that is inserted into the membrane as a homotrimer and cleaved by the matrix metalloprotease TNF converting enzyme to a 51-kDa soluble circulating trimer (soluble TNF-α). Importantly, although it has been demonstrated that, following the onset of ischemic stroke, the expression of TNF-α in the peripheral circulation and central nervous system increases, the effect of TNF-α in the ischemic brain is as of yet unclear [27]. Accordingly, some have demonstrated that increased TNF-α has a deleterious effect in the acute phases of cerebral ischemia [28-30] and that TWEAK-induced cell death is mediated by the interaction between TNF-α and TNF receptor 1 (TNFR1) [31]. In contrast, others have shown that an increase in circulating TNF-α by treatment with either TNF-α or lipopolysaccharide before the onset of the ischemia has a beneficial effect in the ischemic brain and mediates the development of ischemic tolerance [2,32-34].

The extracellular signal-regulated kinases 1 and 2 (ERK 1/2) are members of the family of mitogen-activated protein kinases that have been associated with neurodegeneration and ischemic cell death [35]. However, a growing body of recent evidence indicates that ERK 1/2 activation has a pro-survival effect in the ischemic brain [36], mediated by its ability to attenuate apoptotic cell death [37]. Accordingly, ERK 1/2 mediate the phosphorylation and inactivation of the Bcl-2-associated death promoter protein (BAD). Additionally, ERK 1/2 induce the expression of pro-survival Bcl-2 proteins, notably Bcl-2 and Bcl-xL [38].

Our work indicates that the interaction between TWEAK and Fn14 leads to the development of ischemic tolerance. Indeed, our *in vitro *and *in vivo *data show that either treatment with TWEAK or the induction of endogenous TWEAK and Fn14 expression by sub-lethal hypoxia renders neurons tolerant to a lethal hypoxic and/or ischemic injury. This effect is mediated by TNF-α and ERK 1/2 activation via phosphorylation of BAD. Together, our data reveal a novel mechanism for the development of ischemic tolerance and suggest that treatment with sub-lethal concentrations of TWEAK may be an effective strategy to induce tolerance in the brain of ischemic stroke patients.

## Methods

### Animals and reagents

Murine strains were TWEAK deficient (TWEAK^-/-^) and Fn14 deficient (Fn14^-/-^; kindly provided by Dr. Kyungmin Hahm, Biogen Idec Inc., Cambridge, MA, USA) mice, and TNF-α deficient (TNF-α^-/-^) mice and their wild-type (Wt) littermate controls on a C57BL/6 J genetic background. Other reagents were recombinant TWEAK (rTWEAK; R&D Systems, Minneapolis, MN, USA), the 3-(4,5-dimethylthiazol-2-yl)-2,5-diphenyltetrazolium bromide (MTT) assay (ATCC, Manassas, VA, USA) and the lactate dehydrogenase (LDH) release assay (Roche, Florence, SC, USA), an ELISA kit for TNF-α (Insight Genomics, Falls Church, VA, USA), antibodies against TNF-α and TNFR1 (R&D Systems), ERK 1/2 phosphorylated at Thr202/Tyr204 (pERK), total ERK 1/2 and BAD phosphorylated at Ser112 (pBAD)(Cell Signaling, Danvers, MA, USA), β-actin (Sigma Aldrich, St. Louis, MO, USA), the Mitogen Activated Protein Kinase (MAPK) extracellular signal-regulated kinase (MEK) inhibitor SL327 (Tocris Bioscience, Ellisville, MO, USA), wortmannin, the nuclear markers 4'-6-diamidino-2-phenylindole (DAPI) and triphenyltetrazolium chloride (TTC; Sigma-Aldrich), and the ApopTag Plus Fluorescein In Situ Apoptosis Detection Kit (Chemicon International, Billerica, MA, USA).

### Animal model of cerebral ischemia, *in vivo *model of preconditioning and quantification of the volume of the ischemic lesion

Transient occlusion of the middle cerebral artery (tMCAO) was induced in TWEAK^-/-^, Fn14^-/- ^and TNF-α^-/- ^mice and their corresponding Wt littermate controls with a 6-0 silk suture advanced from the external carotid artery into the internal carotid artery until the origin of the middle cerebral artery (MCA), as described elsewhere [39]. Briefly, animals were anesthetized with 4% chloral hydrate (400 mg/kg intraperitoneal injection) and a nylon monofilament (6-0, Ethicon, Issy Les Moulineaux, France) coated with silicone was introduced through the external carotid artery and advanced up to the origin of the MCA. The suture was withdrawn after 60 minutes of cerebral ischemia. Cerebral perfusion in the distribution of the MCA was monitored throughout the surgical procedure and after reperfusion with a laser Doppler (Perimed Inc., North Royalton, OH, USA), and only animals with a > 70% decrease in cerebral perfusion after occlusion and complete recovery after suture withdrawal were included in this study. The rectal and masseter muscle temperatures were controlled at 37°C with a homoeothermic blanket. Heart rate, systolic, diastolic and mean arterial blood pressures were controlled throughout the surgical procedure with an IITC 229 System (IITC-Lice Science, Woodland Hills, CA, USA). From the total number of mice used in this study (155), 13 (8.3%) were excluded due to incomplete reperfusion after tMCAO and eight (5.16%) died. To induce ischemic tolerance, a subgroup of mice were intraperitoneally injected 24 hours before tMCAO with 0.1 mL of TWEAK (2 mg/mL) alone or in combination with either the MEK inhibitor SL327 (30 mg/kg) or a comparable volume of saline solution. To measure the volume of the ischemic lesion, animals were deeply anesthetized 24 hours after tMCAO, the brains were harvested, cut onto 2 μm sections and stained with TTC. Each section was photographed and the volume of the ischemic lesion was measured by a blinded investigator with the National Institutes of Health Image Analyzer System as described elsewhere [5]. Each observation was repeated ten times. Results are given as a percentage of the stroke volume in untreated animals. All procedures were approved by the Emory University Institutional Animal Care and Use Committee.

### Neuronal cultures, determination of cell survival and death and *in vitro *model of preconditioning

Cerebral cortical neurons were cultured from E16-18 Wt, TWEAK^-/-^, Fn14^-/- ^and TNF-α^-/- ^mice as described elsewhere [40]. Briefly, the cerebral cortex was dissected, transferred into Hanks' balanced salt solution containing 100 units/mL penicillin, 100 μg/mL streptomycin, and 10 mM 4-(2-hydroxyethyl)-1-piperazineethanesulfonic acid, and incubated in trypsin containing 0.02% DNase at 37°C for 15 min. Tissue was then triturated and the supernatant was re-suspended in B27-supplemented neurobasal medium containing 2 mM l-glutamine and plated onto 0.1 mg/mL poly-l-lysine-coated wells.

To study the effect of TWEAK on neuronal survival, Wt cerebral cortical neurons were incubated over 1 or 24 hours with 100 ng/mL or 300 ng/mL of rTWEAK or a comparable volume of vehicle (control), followed 24 hours later by determination of cell survival and/or death with the MTT and LDH release assays following manufacturer's instructions and as described elsewhere [40]. Results are given as a percentage of cell survival or LDH release into the media compared to control cultures. Each experiment was performed in cultures from three different animals and each observation was repeated 15 times.

For TWEAK-induced preconditioning, Fn14^-/-^, TWEAK^-/- ^and Wt cerebral cortical neurons were incubated over 60 minutes with 0 to 300 nM of rTWEAK alone or in combination with antibodies against either TNF-α (0.04 μg/mL) or TNFR1 (100 μg/mL) or an immunoglobulin G isotype control, or with wortmannin 100 nM or SL327 10 μM. Twenty-four hours later, cells were exposed in an anaerobic chamber (Hypoxygen, Frederick, MD, USA) to 55 minutes of oxygen-glucose deprivation (OGD) conditions (< 0.1% oxygen, 94% N_2 _and 5% CO_2 _at 37°C) in glucose-free media containing CaCl_2 _1.8 mM, MgSO_4 _0.8 mM, KCl 5.3 mM, NaHCO_3 _44.05 mM and NaCl 110.34 mM, followed 24 hours later by determination of cell survival and/or death with the MTT and LDH release assays.

To induce hypoxic preconditioning, neurons were exposed to OGD conditions for 30 minutes. A subset of TWEAK^-/- ^and Fn14^-/- ^cells was incubated with rTWEAK 100 ng/mL. The media was then changed to fresh culture media and the cells were returned to the incubator for 24 hours. As controls, sister cultures were kept in OGD media without hypoxia for 30 minutes and then in fresh culture media for 24 hours. After 24 hours, cells were exposed to 55 minutes of OGD conditions and neuronal survival and/or death was studied 24 hours later with the MTT and LDH release assays. Each experiment was performed in cultures from three different animals and each observation was repeated 12 times.

### Quantitative real-time PCR analysis

Wt cerebral cortical neurons were either exposed to 30 minutes of OGD conditions or incubated under normoxic conditions for 60 minutes with TWEAK 100 ng/mL. In both experimental groups the media was changed to fresh culture media and cells were harvested 1, 3 or 6 hours later. Total RNA was isolated using the RNeasy mini kit (Qiagen; Valencia, CA) according to the manufacturer's instructions. Equal amounts of RNA were taken for cDNA synthesis using a High-capacity cDNA Kit (Applied Biosystems). Briefly, 2 × reverse transcription master mix was prepared from 10 × Reverse Transcription Buffer, 25 × deoxyribonucleotide triphosphates, 10 × random primers, and MultiScribe Reverse Transcriptase (Applied Biosystems) and mixed with equal parts of total RNA. The PCRs were performed using TaqMan Gene Expression Assays (Applied Biosystems) using forward and reverse primers as well as internal probes Mm00839900_m1, Mm00489103_m1, Bcl-w Mm00432054_m1 and BclxL Mm00437783_m1 for TWEAK, Fn14, Bcl-w and Bcl-xL, respectively. The PCRs were performed using 7500 Fast Real-Time PCR System (Applied Biosystems) under the following conditions: 50°C for 2 minutes, 95°C for 10 minutes, 40 cycles at 95°C for 15 seconds and 60°C for 1 minute. Each experiment was repeated eight times.

### Determination of TWEAK and TNF-α concentrations

To determine the effect of hypoxia on the release of TWEAK from cerebral cortical neurons, we used an ELISA (Adipobiotech, Santa Clara, CA, USA) to quantify the concentration of TWEAK in the culture media of Wt neurons maintained under normoxic conditions or exposed to 0 to 360 minutes of OGD conditions. Each observation was repeated eight times. To measure the effect of TWEAK on the release of neuronal TNF-α, Wt cerebral cortical neurons were incubated with TWEAK 100 ng/mL or a comparable volume of vehicle (control), followed at 1, 5, 30 or 60 minutes by quantification of TNF-α in the culture media with an ELISA kit (Insight Genomics) following manufacturer's instructions. Each experiment was repeated with neurons cultured from three different animals, and each observation was repeated eight times.

### Western blot analysis

Wt cerebral cortical neurons were incubated for 60 minutes with TWEAK 100 ng/mL alone or in combination with the ERK 1/2 inhibitor 10 μM. After 0 to 180 minutes of incubation, cells were homogenized in radioimmunoprecipitation assay lysis buffer; protein concentration was determined with the bicinchoninic acid protein assay (Thermo Scientific; Canton, GA) and 16 μg of total protein were loaded for SDS-PAGE electrophoresis and immunoblotting with antibodies directed against pERK 1/2, total ERK 1/2, pBAD, total BAD and β-actin. Each observation was repeated four to six times.

### Immunohistochemistry and determination of apoptotic cell death

Wt mice received an intraperitoneal injection of 0.1 mL of TWEAK (2 mg/mL) or a comparable volume of saline solution followed 24 hours later by tMCAO. Twenty-four hours after tMCAO, brains were harvested and 10 μm frozen sections were stained with the ApopTag Plus Fluorecein In Situ Apoptosis Detection Kit following manufacturer's instructions. Briefly, sections were fixed in 1% paraformaldehyde in PBS, pH 7.4, for 10 minutes at room temperature, permeabilized in a 2:1 ratio of ethanol-acetic acid solution for 5 minutes at -20°C, washed twice for 5 minutes and then incubated in a humidified chamber for 1 hour at 37°C with modified nucleotides coupled with the enzyme terminal deoxynucleotidyl transferase. Samples were then washed, incubated with anti-digoxigenin conjugate for 30 minutes and stained with the nuclear marker DAPI.

To quantify the number of terminal deoxynucleotidyl transferase mediated dUTP nick end labeling (TUNEL)-positive cells, each coronal section was divided into 16 square areas (150 mm^2 ^each) that involved the necrotic core and the area of ischemic penumbra, and comparable areas in the non-ischemic hemisphere. Two areas of interest (AOI) were chosen in the boundaries between the ischemic penumbra and necrotic core (AOI-1 that includes the frontoparietal zone and AOI-3 that involves the temporoparietal area), and a third zone was located in the necrotic core (AOI-2). To determine the number of TUNEL-positive cells, images were digitized in a Zeiss Axioplan 2 microscope 20 × objective (Munich, Germany) with a Zeiss AxioCam and imported into AxioVision, viewed at 150% of the original with Image MetaMorph Software and the percentage of TUNEL-positive cells in relation to the total number of DAPI-positive cells per AOI recorded. Each observation was repeated eight times.

To study the effect of TWEAK on pBAD expression, Wt cerebral cortical neurons were incubated for 60 minutes with rTWEAK 100 ng/mL or a comparable volume of vehicle (control) and fixed and stained 1, 3 or 6 hours later with an antibody against pBAD. Each observation was repeated four times.

### Statistical analyses

Data was analyzed by either a Wilcoxon rank-sum test or, in cases where more than one group was compared, by analysis of variance. Statistical significance was determined by *P *< 0.05.

## Results

### The interaction between TWEAK and Fn14 protects neurons from hypoxia-induced cell death

First we used an ELISA to quantify the concentration of TWEAK in the culture media of Wt neurons exposed to OGD conditions for 0 to 6 hours. We found that the concentration of TWEAK in the culture media increased from 6 ± 1.3 pg/mL in cells maintained under normoxic conditions to 10.32 ± 3.64 pg/mL, 14.72 ± 3.47 pg/mL, 13.37 ± 0.7 ng/mL and 6.4 ± 2.5 pg/mL after 30, 60, 180 and 360 minutes of exposure to OGD conditions, respectively (n = 8 per experimental group; *P *< 0.05 when cells exposed to 30, 60 and 180 minutes of OGD were compared to neurons maintained under normoxic conditions; results were non-significant when cells exposed to 6 hours of OGD conditions were compared to control neurons).

**Figure 1:**
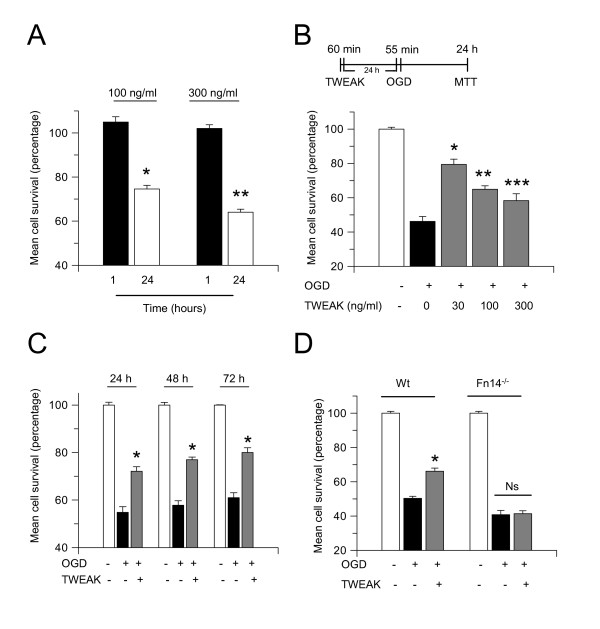
**The interaction between TWEAK and Fn14 induces hypoxic tolerance**. **(A) **Mean cell survival in Wt cerebral cortical neurons incubated 1 or 24 hours with TWEAK 100 ng/mL or 300 ng/mL. *P < 0.05 compared to cells incubated for 60 minutes with TWEAK 100 ng/mL. **P < 0.05 compared to cells incubated for 60 minutes with TWEAK 300 ng/mL. n = 15 per experimental group. Lines denote SD. **(B) **Mean cell survival in Wt cerebral cortical neurons exposed to 55 minutes of OGD conditions 24 hours after 60 minutes of incubation with TWEAK 0 to 300 ng/mL. n = 20 per experimental group; *, ** and *** *P *< 0.05 compared to neurons exposed to 55 minutes of OGD conditions without preconditioning. Lines denote SD. Values are given as cell survival compared to cells exposed to OGD conditions without preconditioning with TWEAK. **(C) **Wt cerebral cortical neurons were preconditioned with TWEAK for 60 minutes 24 hours before exposure to OGD conditions. Mean cell survival was determined with an MTT assay 24, 48 and 72 hours later. n = 12 per experimental group; *P < 0.05 compared to non-preconditioned neurons at each time point. Lines denote SD. **(D) **Mean cell survival in Wt and Fn14^-/- ^cerebral cortical neurons exposed to 55 minutes of OGD conditions 24 hours after 60 minutes of incubation with TWEAK 100 ng/mL. n = 10; *P < 0.05 compared to Wt neurons non-preconditioned with TWEAK. Lines denote SD. Fn14: fibroblast growth factor-inducible 14; Ns: non-significant; OGD: oxygen-glucose deprivation; TWEAK: tumor necrosis factor-like weak inducer of apoptosis; Wt: wild-type.

Activation of inflammatory pathways by a preconditioning stimulus is thought to reduce the inflammatory response to a subsequent period of ischemia, leading to neuroprotection [41], and so we decided to investigate whether the cytokine TWEAK induces hypoxic tolerance. However, because it has been reported that 24 hours of incubation with TWEAK induces neuronal death [18,24], first we investigated whether treatment over a shorter period of time (1 hour) also has an effect on cell survival. Wt cerebral cortical neurons were incubated for 1 or 24 hours with 100 or 300 ng/mL of TWEAK followed by determination of cell survival with the MTT assay as described in the Methods section. We found that, as previously described [24], 24 hours of incubation with 100 or 300 ng/mL of TWEAK is associated with a 25.4% and 37% decrease in neuronal survival, respectively. In contrast, 1 hour of incubation with either 100 or 300 mg/dL of TWEAK did not have an effect on cell survival (Figure 1A; n = 15, *P *< 0.05).

We then used a previously described *in vitro *model of preconditioning [40] to investigate whether treatment with sub-lethal concentrations of TWEAK renders neurons resistant to a subsequent lethal hypoxic injury. Wt cerebral cortical neurons were either left untreated or incubated with TWEAK 0 to 300 ng/mL for 60 minutes followed 24 hours later by exposure to 55 minutes of OGD conditions as depicted in Figure 1B (upper panel) and described in the Methods section. Cell survival was quantified 24 hours later with an MTT assay. Our results indicate that exposure to OGD conditions decreases neuronal survival from 100 ± 0.11% to 46.2 ± 2.8% in non-preconditioned cells. In contrast, cell survival in neurons preconditioned with 30, 100 or 300 ng/mL of TWEAK 24 hours before exposure to OGD conditions was 79.4 ± 3.1%, 64.9 ± 2.0% and 58.3 ± 4%, respectively (Figure 1B; n = 20 per experimental condition; *P *< 0.05 compared to non-preconditioned cells).

To determine whether the protective effect of preconditioning with TWEAK is also observed at later time-points, we quantified cell survival 24, 48 and 72 hours after exposure to 55 minutes of OGD conditions. We found that cell survival decreased from 100 ± 0.8% in control neurons to 54.80 ± 2.4% in neurons exposed to OGD conditions without preconditioning with TWEAK. In contrast, preconditioning with TWEAK 24 hours before exposure to OGD conditions increased neuronal survival to 72.10 ± 1.92%, 77 ± 1.1% and 80 ± 2.0%, when the MTT assay was performed 24, 48 and 72 hours after exposure to OGD, respectively (Figure 1C; n = 12 per experimental condition, *P *< 0.05).

To investigate whether the protective effect of TWEAK is mediated by its interaction with Fn14, we quantified cell survival in Wt and Fn14^-/- ^cerebral cortical neurons incubated with TWEAK 100 ng/mL for 1 hour followed 24 hours later by exposure to 55 minutes of OGD conditions. We found that exposure of non-preconditioned neurons to OGD conditions decreased cell survival from 100 ± 0.2% to 50.3 ± 1.2% in Wt neurons and from 100 ± 0.88% to 40.8 ± 2.4% in Fn14^-/- ^neurons. In contrast, cell survival in Wt and Fn14^-/- ^neurons preconditioned with TWEAK was 66.13 ± 1.8% and 41.3 ± 1.8%, respectively (Figure 1D; n = 10; *P *< 0.05), indicating that genetic deficiency of Fn14 abrogates the ability of TWEAK to induce tolerance to the deleterious effects of OGD.

### Endogenous TWEAK mediates the development of hypoxic and ischemic tolerance

**Figure 2:**
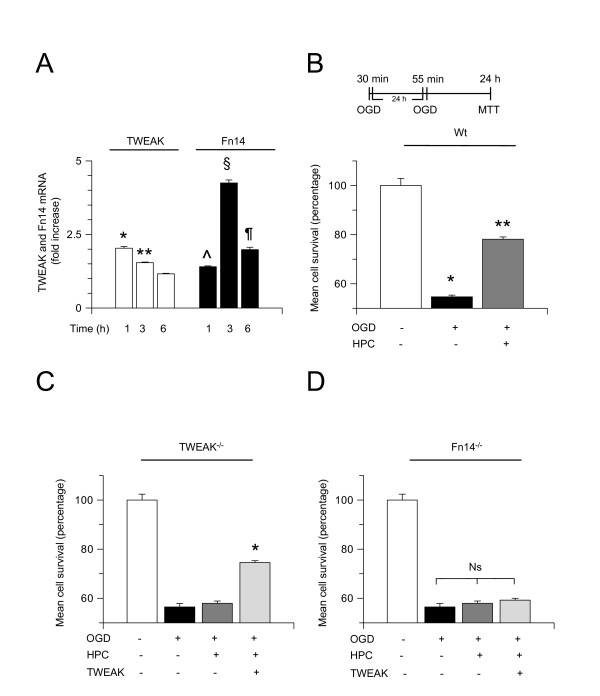
**Endogenous TWEAK mediates the neuroprotective effect of hypoxic preconditioning**. **(A) **Mean fold increase in TWEAK and Fn14 mRNA expression in Wt cerebral cortical neurons 1, 3 and 6 hours after exposure to 30 minutes of OGD (sub-lethal hypoxia). n = 8, * and ** *P *< 0.05 compared to TWEAK mRNA expression in sister cultures maintained under normoxic conditions. ^, § and ¶ *P *< 0.05 compared to Fn14 expression in sister cultures maintained under normoxic conditions. Lines denote SD. (**B-D) **Mean cell survival in Wt (B), TWEAK^-/- ^(C) and Fn14^-/- ^(D) cerebral cortical neurons exposed to 55 minutes of OGD conditions (lethal hypoxia) 24 hours after 30 minutes of exposure to OGD conditions (hypoxic preconditioning, dark gray bars). A subgroup of neurons was exposed to lethal hypoxia without previous preconditioning (black bar). A sub-set of TWEAK^-/- ^and Fn14^-/- ^neurons was incubated with TWEAK 100 ng/mL during the preconditioning phase (hypoxic preconditioning, light gray bars). n = 12 per experimental group; * in B *P *< 0.05 compared to cells maintained under normoxic conditions; ** in B *P <*0.05 compared to neuronal cultures exposed to lethal hypoxia without preconditioning. * in C *P *< 0.05 compared to TWEAK^-/- ^neurons either exposed to lethal OGD without previous preconditioning or preconditioned in the absence of TWEAK. Lines denote SD. Fn14: fibroblast growth factor-inducible 14; HPC: hypoxic preconditioning; MTT: 3-(4,5-dimethylthiazol-2-yl)-2,5-diphenyltetrazolium bromide assay; Ns: non-significant; OGD: oxygen-glucose deprivation; TWEAK: tumor necrosis factor-like weak inducer of apoptosis.

It has been demonstrated that exposure to 30 minutes of OGD conditions (hypoxic preconditioning) not only does not induce cell death [42] but instead renders cerebral cortical neurons tolerant to a lethal hypoxic injury applied at later time points (hypoxic tolerance). Based on these observations we decided to investigate whether endogenous TWEAK plays a role in the protective effect of hypoxic preconditioning. First, we studied the effect of sub-lethal hypoxia (preconditioning) on TWEAK and Fn14 expression. Wt cerebral cortical neurons were exposed to 30 minutes of OGD conditions followed 1, 3 and 6 hours later by quantification of TWEAK and Fn14 mRNA expression by quantitative RT-PCR analysis as described in the Methods section. Our results indicate that sub-lethal hypoxia induces a rapid and transient increase in TWEAK and Fn14 mRNA expression in cerebral cortical neurons that is maximal at 1 hour for TWEAK and 3 hours for Fn14 (Figure 2A).

We then quantified cell survival in Wt, TWEAK^-/- ^and Fn14^-/- ^cerebral cortical neurons exposed to sub-lethal hypoxia (30 minutes of OGD conditions) followed 24 hours later by lethal hypoxia (55 minutes of OGD conditions). Sister cultures were exposed to lethal hypoxia without previous preconditioning as controls. A subgroup of TWEAK^-/- ^and Fn14^-/- ^neurons was incubated with TWEAK 100 ng/mL during the preconditioning phase. Our results indicate that hypoxic preconditioning induces a 23.44% increase in cell survival in Wt neurons (Figure 2B; n = 12, *P *< 0.05) and that this effect is abrogated in neurons genetically deficient in either TWEAK or Fn14 (Figure 2C, D; n = 12). Importantly, incubation with TWEAK during the preconditioning phase had a rescue effect in TWEAK^-/- ^(17.5% increase in neuronal survival) but not in Fn14^-/- ^neurons.

**Figure 3:**
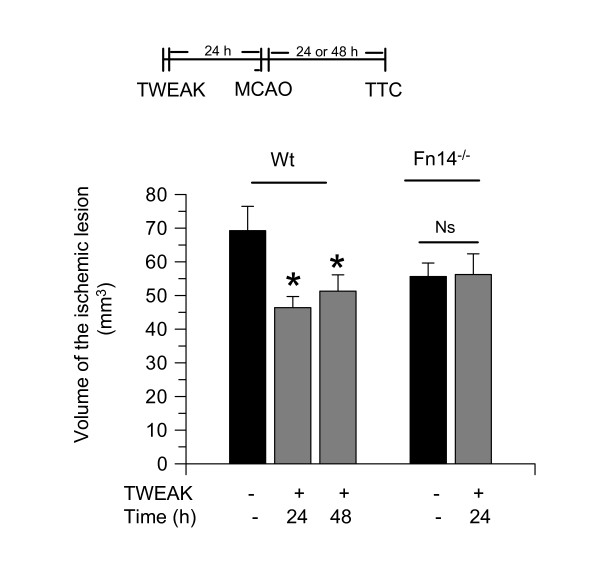
**Treatment with TWEAK induces ischemic tolerance *in vivo***. Wt and Fn14^-/- ^mice treated with vehicle (control; black bars) or TWEAK 0.2 mg by intraperitoneal injection (gray bars) 24 hours before tMCAO. The volume of the ischemic lesion was quantified in Wt mice 24 and 48 hours later. *P < 0.05 compared to non-preconditioned Wt mice. n = 10 per experimental group. Lines denote SD. Fn14: fibroblast growth factor-inducible 14; Ns: non-significant; tMCAO: transient middle cerebral artery occlusion; TTC: triphenyltetrazolium chloride; TWEAK: tumor necrosis factor-like weak inducer of apoptosis; Wt: wild-type.

To investigate whether treatment with TWEAK also has a neuroprotective effect *in vivo*, we measured the volume of the ischemic lesion in Wt and Fn14^-/- ^mice intraperitoneally injected with either TWEAK or a comparable volume of saline solution 24 hours before tMCAO, as described in the Methods section. We found that preconditioning with TWEAK decreases the volume of the ischemic lesion from 69.3 ± 7.2 mm^3 ^to 46.41 ± 3.3 mm^3 ^and 54.31 ± 4.8 mm^3 ^24 and 48 hours after tMCAO, respectively. In contrast, we failed to observe a significant decrease in the volume of the ischemic lesion in Fn14^-/- ^mice preconditioned with TWEAK (Figure 3; n = 10; *P *< 0.05 in Wt mice and non-significant in Fn14^-/- ^animals).

### TNF-α mediates the neuroprotective effect of TWEAK

**Figure 4:**
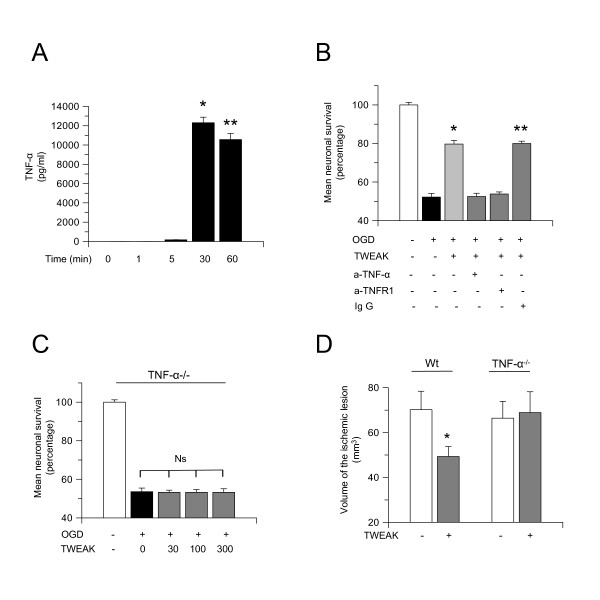
**TNF-α mediates the neuroprotective effect of TWEAK**. **(A) **Mean concentration of TNF-α in the culture media of Wt cerebral cortical neurons 0 to 60 minutes after 60 minutes of incubation with TWEAK 100 ng/mL. n = 8, * and ** *P *< 0.05 compared to untreated sister cultures. Lines denote SD. **(B) **Mean neuronal survival in Wt cerebral cortical neurons exposed to 55 minutes of OGD conditions 24 hours after 60 minutes of incubation with TWEAK 100 ng/mL alone or in the presence of anti-TNF-α 0.04 μg/mL or anti-TNFR1 100 μg/mL antibodies or an immunoglobulin G isotype control. n = 12, *P < 0.05 compared to neurons exposed to OGD conditions without preconditioning with TWEAK, **P < 0.05 compared to cells preconditioned with TWEAK in the presence of anti-TNF-α or anti-TNFR1 antibodies. Lines denote SD. **(C) **Mean neuronal survival in TNF-α^-/- ^neurons incubated for 60 minutes with TWEAK 0 to 300 ng/mL 24 hours before exposure to 55 minutes of OGD conditions. n = 10 per group. **(D) **Volume of the ischemic lesion in Wt and TNF-α^-/- ^mice treated with vehicle control (white bars) or TWEAK 0.2 mg by intraperitoneal injection (gray bars) 24 hours before tMCAO. *P < 0.05 compared to non-preconditioned Wt mice. n = 12 per experimental group. Lines denote SD. Ig G: immunoglobulin G; Ns: non-significant; OGD: oxygen-glucose deprivation; TNF: tumor necrosis factor; TNFR1: tumor necrosis factor receptor 1; TWEAK: tumor necrosis factor-like weak inducer of apoptosis; Wt: wild-type.

Because it has been reported that TNF-α mediates some of the biological effects of TWEAK [43], we investigated whether TNF-α also mediates TWEAK-induced tolerance. First, we used an ELISA to study the expression of TNF-α in the culture media of Wt cerebral cortical neurons incubated for 1 to 60 minutes with TWEAK 100 ng/mL. Our results indicated that TWEAK induces a rapid increase in the expression of neuronal TNF-α and that this effect is maximum at 30 minutes of incubation (Figure 4A; 12,309 ± 526 pg/mL, n = 8, *P *< 0.05).

We then used the MTT assay to study cell survival in Wt cerebral cortical neurons incubated for 60 minutes with TWEAK 100 ng/mL alone or in combination with neutralizing antibodies against either TNF-α 0.04 μg/mL or TNFR1 100 μg/mL, or an immunoglobulin G isotype control, followed 24 hours later by exposure to 55 minutes of OGD conditions (lethal hypoxia). We found that, whereas treatment with TWEAK rendered neurons tolerant to a lethal hypoxia insult applied 24 hours later, this preconditioning effect was abrogated by incubation with either anti-TNF-α or anti-TNFR1 antibodies (Figure 4B; n = 12). To further study the role of TNF-α on TWEAK-induced neuroprotection, we quantified cell survival in TNF-α^-/- ^cerebral cortical neurons incubated for 1 hour with TWEAK 0 to 300 ng/mL followed 24 hours later by exposure to 55 minutes of OGD conditions. Our results indicated that TWEAK fails to induce hypoxic tolerance in TNF-α^-/- ^neurons (Figure 4C: n = 10, non-significant).

To investigate whether TNF-α also mediates the protective effect of treatment with TWEAK *in vivo*, we measured the volume of the ischemic lesion in Wt and TNF-α^-/- ^mice intraperitoneally injected with either TWEAK or a comparable volume of saline solution 24 hours before tMCAO. We found that, whereas preconditioning with TWEAK decreases the volume of the ischemic lesion in Wt mice from 70.5 ± 8.2 mm^3 ^to 49.35 ± 4.4 mm^3 ^in Wt mice, this effect is abrogated by a genetic deficiency of TNF-α (Figure 4D; n = 12).

### The ability of TWEAK to induce ischemic tolerance is mediated by activation of the ERK 1/2

**Figure 5:**
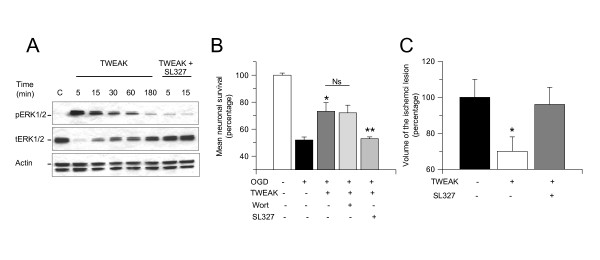
**ERK 1/2 mediates the TWEAK-induced hypoxic and/or ischemic tolerance**. **(A) **Representative western blot analysis for pERK 1/2 in Wt cerebral cortical neurons incubated for 0 to 180 minutes with TWEAK 100 ng/mL alone or in combination with SL327 10 μM. **(B) **Mean neuronal survival in Wt cerebral cortical neurons exposed to 55 minutes of oxygen-glucose deprivation conditions 24 hours after 60 minutes of incubation with TWEAK 100 ng/mL alone (white bar) or in combination with either wortmannin 100 nM (dark gray bar) or the ERK 1/2 inhibitor SL327 (light gray bar). n = 16 per group, *P < 0.05 compared to neurons maintained under normoxic conditions, **P < 0.05 compared to neurons preconditioned with TWEAK alone. Lines denote SD. **(C) **Volume of the ischemic lesion in Wt mice treated with vehicle (control, black bar) or TWEAK 0.2 mg alone (white bar) or in combination with the ERK 1/2 inhibitor SL327 10 uM (gray bar) 24 hours before tMCAO. *P < 0.05 compared to non-preconditioned mice and with mice preconditioned with TWEAK alone. n = 8 per experimental group. Lines denote SD. Ns: non-significant; OGD: oxygen-glucose deprivation; pERK: ERK 1/2 phosphorylated at Thr202/Tyr204; tERK: total ERK; tMCAO: transient middle cerebral artery occlusion; TWEAK: tumor necrosis factor-like weak inducer of apoptosis; Wort: wortmannin; Wt: wild-type.

Because ERK 1/2 mediates the protective effects of several factors that enhance neuronal survival following exposure to hypoxia/ischemia [35], we investigated whether ERK 1/2 also mediates the neuroprotective effect of TWEAK. First, we studied the expression of pERK 1/2 in Wt cerebral cortical neurons incubated for 0 to180 minutes with TWEAK 100 ng/mL. We found that TWEAK induces ERK 1/2 activation, and that this effect is maximal at 5 to 15 minutes of incubation (Figure 5A).

To investigate whether TWEAK-induced hypoxic tolerance is mediated by ERK 1/2 activation, we quantified cell survival in Wt cerebral cortical neurons exposed to 55 minutes of OGD conditions 24 hours after 1 hour of incubation with TWEAK 100 ng/mL either alone or in combination with the ERK 1/2 inhibitor SL327 10 μM. Our results indicate that the preconditioning effect of TWEAK is abrogated by ERK 1/2 inhibition (Figure 5B; n = 12).

Activation of the PI3K/Akt pathway also promotes survival in neurons exposed to hypoxic conditions [40], and so we investigated the effect of PI3K inhibition with wortmannin 20 nM on TWEAK-induced preconditioning. We found that inhibition of the PI3K/Akt pathway does not abrogate the neuroprotective effect of TWEAK (Figure 5B).

To determine whether the protective effect observed following treatment with TWEAK *in vivo *was also mediated by ERK 1/2 activation we measured the volume of the ischemic lesion in Wt mice intraperitoneally injected with TWEAK, alone or in combination with blood-brain barrier-permeable ERK 1/2 inhibitor SL327, 24 hours before tMCAO. Our results indicate that the beneficial effect of preconditioning with TWEAK *in vivo *is abrogated by ERK 1/2 inhibition (Figure 5D; n = 8).

### Preconditioning with TWEAK attenuates cerebral ischemia-induced apoptotic cell death

Because experimental work with glial cell tumors indicates that TWEAK induces the expression of the anti-apoptotic proteins Bcl-xL and Bcl-w, we used RT-PCR analysis to study Bcl-xL and Bcl-w mRNA expression in Wt cerebral cortical neurons incubated with TWEAK 1, 3, 6 or 24 hours. Our data indicated that TWEAK does not induce the expression of Bcl-xL and Bcl-w in neurons (data not shown).

**Figure 6:**
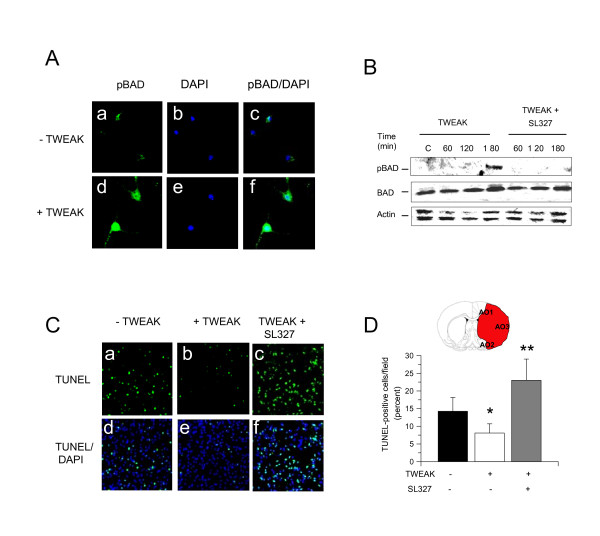
**Effect of preconditioning with TWEAK on cerebral ischemia-induced cell death**. **(A) **Representative micrographs of Wt cerebral cortical neurons immunostained for pBAD 3 hours after 60 minutes of treatment with either vehicle (control; panels a-c) or TWEAK 100 ng/mL (panels d-f). Green is pBAD, blue is DAPI. Magnification × 40. **(B) **Representative western blot analysis for pBAD and total BAD expression 0 to 180 minutes after 60 minutes of incubation with TWEAK alone or in combination with SL327 10 μM. **(C) **Representative micrographs of TUNEL staining in area of AOI2 in Wt mice intraperitoneally injected with saline solution (panels a and b), TWEAK 0.2 mg (panels b and c), or with a combination of TWEAK and SL327 (panels c and f) 24 hours before tMCAO. Green is TUNEL, blue is DAPI. Magnification × 20. **(D) **Diagram showing the three different AOIs and percentage of TUNEL-positive cells per field AOI 1, 2 and 3 in the ischemic tissue of Wt mice intraperitoneally injected with saline solution (black bar), TWEAK 0.2 mg (white bar), or TWEAK plus SL327 24 hours before tMCAO. n = 10, *P < 0.05 compared to untreated mice. Lines depict SD. AOI: area of interest; BAD: Bcl-2-associated death promoter protein; DAPI: 4'-6-diamidino-2-phenylindole; pBAD: BAD phosphorylated at Ser112; TUNEL: terminal deoxynucleotidyl transferase mediated dUTP nick end labeling; TWEAK: tumor necrosis factor-like weak inducer of apoptosis.

It has been demonstrated that ERK 1/2 induces the phosphorylation of BAD at Ser^112^. Because our data indicate that the protective effect of TWEAK is mediated by ERK 1/2 activation, then we investigated the effect of TWEAK on BAD phosphorylation. To test this hypothesis we studied the expression of pBAD 1, 3 and 6 hours after 60 minutes of incubation with TWEAK 100 ng/mL. Our results indicated that TWEAK induces rapid phosphorylation of BAD in cerebral cortical neurons (Figure 6A).

To further characterize these results, we studied the expression of pBAD in Wt cerebral cortical neurons incubated with TWEAK alone or in combination with SL327. We found that TWEAK induced pBAD expression in neurons and that this effect is inhibited by co-treatment with SL327 (Figure 6B).

Because phosphorylation of BAD has an anti-apoptotic effect, we investigated whether preconditioning with TWEAK decreases cerebral ischemia-induced apoptotic cell death. Wt mice were intraperitoneally injected with TWEAK or a comparable volume of saline solution, followed 24 hours later by tMCAO and determination of apoptotic cell death in the ischemic tissue as described in the Methods section. We found that preconditioning with TWEAK decreases the percentage of TUNEL-positive cells per field in the ischemic area from 14.25 ± 3.9% in saline solution-treated animals to 8.1 ± 2.3% in animals pre-treated with TWEAK (Figure 6C, D; n = 10, *P *< 0.05). Importantly, co-treatment with SL327 not only abrogated the effect of TWEAK on apoptotic cell death but also increased the number of apoptotic cells per field to 23 ± 6% (Figure 6D; n = 10, *P *< 0.05).

## Discussion

Ischemic stroke has a devastating effect on the brain. Indeed, one minute of cerebral ischemia destroys approximately 1.9 million neurons and 14 billion synapses [44]. However, despite this appalling outcome, the brain has the ability to develop tolerance to a lethal hypoxic and/or ischemic injury, suggesting the existence of a mechanism to adapt to hypoxic and ischemic conditions. Thus, elucidating the mechanisms underlying the development of ischemic tolerance may lead to the development of an effective neuroprotective tool to protect the brain from the harmful effects of ischemic stroke.

Our data indicates that the interaction between the cytokine TWEAK and its receptor Fn14 renders neurons tolerant to a lethal hypoxic and/or ischemic injury. This suggests that, as also described with other signaling pathways, TWEAK/Fn14 induces the acquisition of resistance against hypoxic and/or ischemic damage. Indeed, our data indicate that although TWEAK is able to induce neuronal death, low level or short exposure to TWEAK induces ischemic tolerance, as do other noxious stimuli below the threshold of significant tissue damage.

The onset of cerebral ischemia is followed by an inflammatory reaction that has been commonly linked with cell death and poor neurological outcome. However, a growing body of evidence indicates that the development of a proinflammatory status may also have a beneficial effect in the ischemic brain. Indeed, is now well recognized that regardless of the preconditioning stimulus, the development of ischemic tolerance is not associated with variations in regional tissue perfusion, but instead with cellular changes triggered by proinflammatory cytokines [41]. In agreement with these observations, our data show that treatment with TWEAK induces ischemic tolerance *in vivo *and *in vitro *and that a genetic deficiency of either TWEAK or Fn14 abrogates the beneficial effects of preconditioning with sub-lethal hypoxia (hypoxic preconditioning).

In apparent contradiction with a neuroprotective role for TWEAK are the reports that the interaction between TWEAK and Fn14 induces cell death. Accordingly, earlier studies indicate that a genetic deficiency of TWEAK or Fn14 [23], or treatment with either monoclonal antibodies against TWEAK [18] or with a soluble Fn14-Fc-decoy receptor [5] are associated with improved neurological outcome following experimental cerebral ischemia. However, in most of the cases the effect of TWEAK on cell death is relatively weak and requires long incubation periods [21]. These observations agree with our results, which indicate that 24 hours but not 1 hour of incubation with TWEAK induces neuronal death. Importantly, our data show that sub-lethal hypoxia (hypoxic preconditioning) has a rapid and transient effect on TWEAK and Fn14 expression in cerebral cortical neurons, suggesting that the pro-survival or death promoting effects of TWEAK are associated with a transient or sustained increase in the expression of this cytokine, respectively. Together, these data indicate that TWEAK and Fn14 have a dual role in the central nervous system.

A pro-survival effect of TWEAK is supported by work from other groups with glial cell tumors demonstrating that TWEAK suppresses apoptotic cell death in glioma via its ability to induce Bcl-xL and/or Bcl-w expression [26]. Our data indicate that although TWEAK does not induce Bcl-xL and/or Bcl-w expression in cerebral cortical neurons, it causes a rapid increase in BAD phosphorylation at Ser^112^, inhibiting its pro-apoptotic properties.

It has been described that ERK 1/2 mediates BAD phosphorylation at Ser^112 ^[45]. Our results show that TWEAK induces ERK 1/2 activation, and that ERK 1/2 inhibition abrogates the beneficial effect of TWEAK. Importantly, in contrast with the observation that the pro-survival effect of ERK 1/2 is associated with activation of the PI3K/Akt pathway [37], our data show that treatment with wortmannin does not inhibit TWEAK-induced neuroprotection, suggesting the existence of an alternative pathway for TWEAK-induced ERK 1/2-mediated ischemic tolerance.

There are two TNF-α receptors: TNFR1 (p55) and TNFR2 (p75). TNFR1 has an intracellular death domain sequence. Accordingly, the interaction between TNF-α and TNFR1 has been linked with cell death in different *in vivo *and *in vitro *models of neurodegeneration [27]. However, in apparent discrepancy with these observations, animals genetically deficient in TNFR1 have a worse neurological outcome following experimental cerebral ischemia than their wild-type controls [46]. Additionally, later studies indicate that the interaction between TNF-α and TNFR1 induces tolerance in the ischemic brain, and that this effect is mediated by erythropoietin and vascular endothelial growth factor [47]. In line with these observations, our results show that the ability of TWEAK to induce ischemic tolerance is abrogated by a genetic deficiency of TNF-α or TNFR1 antagonism.

## Conclusions

Based on our data, we propose a model where, in response to sub-lethal hypoxia and/or ischemia, the interaction between TWEAK and Fn14 promotes the development of ischemic tolerance via TNF-α and ERK 1/2-mediated inhibition of apoptotic cell death. Our results also suggest that treatment with TWEAK may be a therapeutic strategy to protect the brain of patients at high risk of ischemic stroke.

## Abbreviations

AOI: area of interest; BAD: Bcl-2-associated death promoter protein; Bcl-2: B-cell lymphoma 2; DAPI: 4'-6-diamidino-2-phenylindole; ELISA: enzyme-linked immunosorbent assay; ERK 1/2: extracellular signal-regulated kinases 1 and 2; Fc: crystallizable fragment; Fn14: fibroblast growth factor-inducible 14; kDA: kiloDaltons; LDH: lactate dehydrogenase; MCA: middle cerebral artery: MTT: 3-(4,5-dimethylthiazol-2-yl)-2,5-diphenyltetrazolium bromide; OGD: oxygen-glucose deprivation; PBS: phosphate-buffered saline; PCR: polymerase chain reaction; pBAD: BAD phosphorylated at Ser112; pERK 1/2: ERK 1/2 phosphorylated at Thr202/Tyr204; RT: reverse transcriptase; tMCAO: transient middle cerebral artery occlusion; TNF: tumor necrosis factor; TNFR1: tumor necrosis factor receptor 1; TTC: triphenyltetrazolium chloride; TUNEL: terminal deoxynucleotidyl transferase mediated dUTP nick end labeling; TWEAK: tumor necrosis factor-like weak inducer of apoptosis; Wt; wild-type.

## Competing interests

The authors declare that they have no competing interests.

## Authors' contributions

RE, FW, WH and JW performed experiments. MY designed experiments, supervised their performance and wrote the paper. All authors read and approved the final manuscript.

## References

[B1] Kirino T Nakagomi T Kanemitsu H Tamura A Ischemic tolerance Adv Neurol 1996 71 505 511 8790824

[B2] Kirino T Ischemic tolerance J Cereb Blood Flow Metab 2002 22 1283 1296 12439285 10.1097/01.WCB.0000040942.89393.88

[B3] Roger VL Go AS Lloyd-Jones DM Adams RJ Berry JD Brown TM Carnethon MR Dai S De SG Ford ES Fox CS Fullerton HJ Gillespie C Greenlund KJ Hailpern SM Heit JA Michael HP Howard VJ Kissela BM Kittner SJ Lackland DT Lichtman JH Lisabeth LD Makuc DM Marcus GM Marelli A Matchar DB McDermott MM Meigs JB Moy CS Mozaffarian D Mussolino ME Nichol G Paynter NP Rosamond WD Sorlie PD Stafford RS Turan TN Turner MB Wong ND Wylie-Rosett J Roger VL Turner MB Executive summary: heart disease and stroke statistics-2011 update: a report from the American heart association Circulation 2011 123 459 463 10.1161/CIR.0b013e3182009701 PMC4418670 21160056

[B4] Chicheportiche Y Bourdon PR Xu H Hsu YM Scott H Hession C Garcia I Browning JL TWEAK, a new secreted ligand in the tumor necrosis factor family that weakly induces apoptosis J Biol Chem 1997 272 32401 32410 10.1074/jbc.272.51.32401 9405449

[B5] Yepes M Brown SA Moore EG Smith EP Lawrence DA Winkles JA A soluble Fn14-Fc decoy receptor reduces infarct volume in a murine model of cerebral ischemia Am J Pathol 2005 166 511 520 10.1016/S0002-9440(10)62273-0 15681834 PMC1602337

[B6] Yepes M Tweak and FN14 in central nervous system health and disease Front Biosci 2007 12 2772 2781 17485258 10.2741/2271

[B7] Wiley SR Cassiano L Lofton T Davis-Smith T Winkles JA Lindner V Liu H Daniel TO Smith CA Fanslow WC A novel TNF receptor family member binds TWEAK and is implicated in angiogenesis Immunity 2001 15 837 846 10.1016/S1074-7613(01)00232-1 11728344

[B8] Lynch CN Wang YC Lund JK Chen YW Leal JA Wiley SR TWEAK induces angiogenesis and proliferation of endothelial cells J Biol Chem 1999 274 8455 8459 10.1074/jbc.274.13.8455 10085077

[B9] Harada N Nakayama M Nakano H Fukuchi Y Yagita H Okumura K Pro-inflammatory effect of TWEAK/Fn14 interaction on human umbilical vein endothelial cells Biochem Biophys Res Commun 2002 299 488 493 10.1016/S0006-291X(02)02670-0 12445828

[B10] Donohue PJ Richards CM Brown SA Hanscom HN Buschman J Thangada S Hla T Williams MS Winkles JA TWEAK is an endothelial cell growth and chemotactic factor that also potentiates FGF-2 and VEGF-A mitogenic activity Arterioscler Thromb Vasc Biol 2003 23 594 600 10.1161/01.ATV.0000062883.93715.37 12615668

[B11] Tran NL McDonough WS Donohue PJ Winkles JA Berens TJ Ross KR Hoelzinger DB Beaudry C Coons SW Berens ME The human Fn14 receptor gene is up-regulated in migrating glioma cells in vitro and overexpressed in advanced glial tumors Am J Pathol 2003 162 1313 1321 10.1016/S0002-9440(10)63927-2 12651623 PMC1851233

[B12] Polek TC Talpaz M Darnay BG Spivak-Kroizman T TWEAK mediates signal transduction and differentiation of RAW264.7 cells in the absence of Fn14/TweakR. Evidence for a second TWEAK receptor J Biol Chem 2003 278 32317 32323 10.1074/jbc.M302518200 12794080

[B13] Saas P Boucraut J Walker PR Quiquerez AL Billot M Desplat-Jego S Chicheportiche Y Dietrich PY TWEAK stimulation of astrocytes and the proinflammatory consequences Glia 2000 32 102 107 10.1002/1098-1136(200010)32:1<102::AID-GLIA100>3.0.CO;2-U 10975915

[B14] Chicheportiche Y Chicheportiche R Sizing I Thompson J Benjamin CB Ambrose C Dayer JM Proinflammatory activity of TWEAK on human dermal fibroblasts and synoviocytes: blocking and enhancing effects of anti-TWEAK monoclonal antibodies Arthritis Res 2002 4 126 133 10.1186/ar388 11879548 PMC83846

[B15] Xu H Okamoto A Ichikawa J Ando T Tasaka K Masuyama K Ogawa H Yagita H Okumura K Nakao A TWEAK/Fn14 interaction stimulates human bronchial epithelial cells to produce IL-8 and GM-CSF Biochem Biophys Res Commun 2004 318 422 427 10.1016/j.bbrc.2004.04.036 15120617

[B16] Kim SH Kang YJ Kim WJ Woo DK Lee Y Kim DI Park YB Kwon BS Park JE Lee WH TWEAK can induce pro-inflammatory cytokines and matrix metalloproteinase-9 in macrophages Circ J 2004 68 396 399 10.1253/circj.68.396 15056843

[B17] Jin L Nakao A Nakayama M Yamaguchi N Kojima Y Nakano N Tsuboi R Okumura K Yagita H Ogawa H Induction of RANTES by TWEAK/Fn14 interaction in human keratinocytes J Invest Dermatol 2004 122 1175 1179 10.1111/j.0022-202X.2004.22419.x 15140220

[B18] Potrovita I Zhang W Burkly L Hahm K Lincecum J Wang MZ Maurer MH Rossner M Schneider A Schwaninger M Tumor necrosis factor-like weak inducer of apoptosis-induced neurodegeneration J Neurosci 2004 24 8237 8244 10.1523/JNEUROSCI.1089-04.2004 15385607 PMC6729692

[B19] Haile WB Echeverry R Wu J Yepes M The interaction between tumor necrosis factor-like weak inducer of apoptosis and its receptor fibroblast growth factor-inducible 14 promotes the recruitment of neutrophils into the ischemic brain J Cereb Blood Flow Metab 2010 30 1147 1156 10.1038/jcbfm.2009.280 20068578 PMC2949208

[B20] Inta I Frauenknecht K Dorr H Kohlhof P Rabsilber T Auffarth GU Burkly L Mittelbronn M Hahm K Sommer C Schwaninger M Induction of the cytokine TWEAK and its receptor Fn14 in ischemic stroke J Neurol Sci 2008 275 117 120 10.1016/j.jns.2008.08.005 18793781

[B21] Winkles JA The TWEAK-Fn14 cytokine-receptor axis: discovery, biology and therapeutic targeting Nat Rev Drug Discov 2008 7 411 425 10.1038/nrd2488 18404150 PMC3018765

[B22] Schneider A Martin-Villalba A Weih F Vogel J Wirth T Schwaninger M NF-kappaB is activated and promotes cell death in focal cerebral ischemia Nat Med 1999 5 554 559 10.1038/8432 10229233

[B23] Zhang X Winkles JA Gongora MC Polavarapu R Michaelson JS Hahm K Burkly L Friedman M Li XJ Yepes M TWEAK-Fn14 pathway inhibition protects the integrity of the neurovascular unit during cerebral ischemia J Cereb Blood Flow Metab 2007 27 534 544 10.1038/sj.jcbfm.9600368 16835630

[B24] Haile WB Echeverry R Wu F Guzman J An J Wu J Yepes M Tumor necrosis factor-like weak inducer of apoptosis and fibroblast growth factor-inducible 14 mediate cerebral ischemia-induced poly(ADP-ribose) polymerase-1 activation and neuronal death Neuroscience 2010 171 1256 1264 10.1016/j.neuroscience.2010.10.029 20955770 PMC2991428

[B25] Nakayama M Ishidoh K Kayagaki N Kojima Y Yamaguchi N Nakano H Kominami E Okumura K Yagita H Multiple pathways of TWEAK-induced cell death J Immunol 2002 168 734 743 11777967 10.4049/jimmunol.168.2.734

[B26] Tran NL McDonough WS Savitch BA Sawyer TF Winkles JA Berens ME The tumor necrosis factor-like weak inducer of apoptosis (TWEAK)-fibroblast growth factor-inducible 14 (Fn14) signaling system regulates glioma cell survival via NFkappaB pathway activation and BCL-XL/BCL-W expression J Biol Chem 2005 280 3483 3492 15611130 10.1074/jbc.M409906200

[B27] Hallenbeck JM The many faces of tumor necrosis factor in stroke Nat Med 2002 8 1363 1368 10.1038/nm1202-1363 12457181

[B28] Barone FC Arvin B White RF Miller A Webb CL Willette RN Lysko PG Feuerstein GZ Tumor necrosis factor-alpha. A mediator of focal ischemic brain injury Stroke 1997 28 1233 1244 10.1161/01.STR.28.6.1233 9183357

[B29] Dawson DA Martin D Hallenbeck JM Inhibition of tumor necrosis factor-alpha reduces focal cerebral ischemic injury in the spontaneously hypertensive rat Neurosci Lett 1996 218 41 44 10.1016/0304-3940(96)13116-5 8939476

[B30] Nawashiro H Martin D Hallenbeck JM Neuroprotective effects of TNF binding protein in focal cerebral ischemia Brain Res 1997 778 265 271 10.1016/S0006-8993(97)00981-5 9459543

[B31] Schneider P Schwenzer R Haas E Muhlenbeck F Schubert G Scheurich P Tschopp J Wajant H TWEAK can induce cell death via endogenous TNF and TNF receptor 1 Eur J Immunol 1999 29 1785 1792 10.1002/(SICI)1521-4141(199906)29:06<1785::AID-IMMU1785>3.0.CO;2-U 10382740

[B32] Tasaki K Ruetzler CA Ohtsuki T Martin D Nawashiro H Hallenbeck JM Lipopolysaccharide pre-treatment induces resistance against subsequent focal cerebral ischemic damage in spontaneously hypertensive rats Brain Res 1997 748 267 270 10.1016/S0006-8993(96)01383-2 9067475

[B33] Ginis I Schweizer U Brenner M Liu J Azzam N Spatz M Hallenbeck JM TNF-alpha pretreatment prevents subsequent activation of cultured brain cells with TNF-alpha and hypoxia via ceramide Am J Physiol 1999 276 C1171 C1183 10329967 10.1152/ajpcell.1999.276.5.C1171

[B34] Nawashiro H Tasaki K Ruetzler CA Hallenbeck JM TNF-alpha pretreatment induces protective effects against focal cerebral ischemia in mice J Cereb Blood Flow Metab 1997 17 483 490 9183285 10.1097/00004647-199705000-00001

[B35] Irving EA Bamford M Role of mitogen- and stress-activated kinases in ischemic injury J Cereb Blood Flow Metab 2002 22 631 647 12045661 10.1097/00004647-200206000-00001

[B36] Nozaki K Nishimura M Hashimoto N Mitogen-activated protein kinases and cerebral ischemia Mol Neurobiol 2001 23 1 19 10.1385/MN:23:1:01 11642541

[B37] Hetman M Gozdz A Role of extracellular signal regulated kinases 1 and 2 in neuronal survival Eur J Biochem 2004 271 2050 2055 10.1111/j.1432-1033.2004.04133.x 15153093

[B38] Boucher MJ Morisset J Vachon PH Reed JC Laine J Rivard N MEK/ERK signaling pathway regulates the expression of Bcl-2, Bcl-X(L), and Mcl-1 and promotes survival of human pancreatic cancer cells J Cell Biochem 2000 79 355 369 10.1002/1097-4644(20001201)79:3<355::AID-JCB20>3.0.CO;2-0 10972974

[B39] Belayev L Busto R Zhao W Fernandez G Ginsberg MD Middle cerebral artery occlusion in the mouse by intraluminal suture coated with poly-L-lysine: neurological and histological validation Brain Res 1999 833 181 190 10.1016/S0006-8993(99)01528-0 10375693

[B40] Echeverry R Wu J Haile WB Guzman J Yepes M Tissue-type plasminogen activator is a neuroprotectant in the mouse hippocampus J Clin Invest 2010 120 2194 2205 10.1172/JCI41722 20440070 PMC2877952

[B41] Kariko K Weissman D Welsh FA Inhibition of toll-like receptor and cytokine signaling--a unifying theme in ischemic tolerance J Cereb Blood Flow Metab 2004 24 1288 1304 15545925 10.1097/01.WCB.0000145666.68576.71

[B42] Grabb MC Choi DW Ischemic tolerance in murine cortical cell culture: critical role for NMDA receptors J Neurosci 1999 19 1657 1662 10024352 10.1523/JNEUROSCI.19-05-01657.1999 PMC6782179

[B43] Nakayama M Ishidoh K Kojima Y Harada N Kominami E Okumura K Yagita H Fibroblast growth factor-inducible 14 mediates multiple pathways of TWEAK-induced cell death J Immunol 2003 170 341 348 12496418 10.4049/jimmunol.170.1.341

[B44] Saver JL Time is brain--quantified Stroke 2006 37 263 266 16339467 10.1161/01.STR.0000196957.55928.ab

[B45] Fueller J Becker M Sienerth AR Fischer A Hotz C Galmiche A C-RAF activation promotes BAD poly-ubiquitylation and turn-over by the proteasome Biochem Biophys Res Commun 2008 370 552 556 10.1016/j.bbrc.2008.03.141 18402774

[B46] Gary DS Bruce-Keller AJ Kindy MS Mattson MP Ischemic and excitotoxic brain injury is enhanced in mice lacking the p55 tumor necrosis factor receptor J Cereb Blood Flow Metab 1998 18 1283 1287 9850139 10.1097/00004647-199812000-00001

[B47] Taoufik E Petit E Divoux D Tseveleki V Mengozzi M Roberts ML Valable S Ghezzi P Quackenbush J Brines M Cerami A Probert L TNF receptor I sensitizes neurons to erythropoietin- and VEGF-mediated neuroprotection after ischemic and excitotoxic injury Proc Natl Acad Sci USA 2008 105 6185 6190 10.1073/pnas.0801447105 18413601 PMC2299225

